# Copper deficiency alters shoot architecture and reduces fertility of both gynoecium and androecium in *Arabidopsis thaliana*

**DOI:** 10.1002/pld3.288

**Published:** 2020-11-29

**Authors:** Maryam Rahmati Ishka, Olena K. Vatamaniuk

**Affiliations:** ^1^ Soil and Crop Sciences Section School of Integrative Plant Science Cornell University Ithaca NY USA; ^2^ Plant Biology Section School of Integrative Plant Science Cornell University Ithaca NY USA

**Keywords:** Arabidopsis, auxin, copper deficiency, fertility, pollen, stigma

## Abstract

Copper deficiency reduces plant growth, male fertility, and seed set. The contribution of copper to female fertility and the underlying molecular aspects of copper deficiency‐caused phenotypes are not well known. We show that among copper deficiency‐caused defects in *Arabidopsis thaliana* were also the increased shoot branching, delayed flowering and senescence, and entirely abolished gynoecium fertility. The increased shoot branching of copper‐deficient plants was rescued by the exogenous application of auxin or copper. The delayed flowering was associated with the decreased expression of the floral activator, *FT*. Copper deficiency also decreased the expression of senescence‐associated genes, *WRKY53* and *SAG13*, but increased the expression of *SAG12*. The reduced fertility of copper‐deficient plants stemmed from multiple factors including the abnormal stigma papillae development, the abolished gynoecium fertility, and the failure of anthers to dehisce. The latter defect was associated with reduced lignification, the upregulation of copper microRNAs and the downregulation of their targets, laccases, implicated in lignin synthesis. Copper‐deficient plants accumulated ROS in pollen and had reduced cytochrome *c* oxidase activity in both leaves and floral buds. This study opens new avenues for the investigation into the relationship between copper homeostasis, hormone‐mediated shoot architecture, gynoecium fertility, and copper deficiency‐derived nutritional signals leading to the delay in flowering and senescence.

## 1 INTRODUCTION

It has been known for decades that the deficiency in the micronutrient copper in alkaline soils compromises plant fertility with the most negative impact on wheat grain production (Broadley et al., 2012; Graham, 1978; Graves & Sutcliffe, 1974; Shorrocks & Alloway, 1988; Solberg et al., 1999). In‐line with the essential role of copper in plant reproduction, recent studies using synchrotron x‐ray fluorescence microscopy have shown that the bulk of copper is associated with anthers and pistils in *Arabidopsis thaliana* and *Brachypodium distachyon* and failure to transport copper to reproductive structures in these species and *Oryza sativa* significantly reduces fertility (Sheng et al., 2019; Yan et al., 2017; Zhang, Lu, et al., 2018).

The essential role of copper stems from its involvement in redox reactions and its indispensable role as a cofactor for more than 100 enzymes including those participating in important biological processes such as respiration, photosynthesis, and scavenging of reactive oxidative species (ROS) (Broadley et al., 2012; Burkhead et al., 2009; Ravet & Pilon, 2013). It is noteworthy that because copper is redox‐active, an increased concentration of free copper ions causes cellular toxicity. To maintain copper homeostasis, plants regulate copper uptake by transcriptional and post‐transcriptional regulation of copper transporters and *via* economizing on copper during deficiency (Burkhead et al., 2009; Ravet & Pilon, 2013). The regulation of copper uptake and internal distribution include transporters from CTR/COPT and Yellow Stripe‐like (YSL) families (Burkhead et al., 2009). Copper economy/metal switch mechanism involves the increased expression of copper‐responsive microRNAs, including *miR397*, *miR398*, *miR408,* and *miR857* that, in turn, facilitates the mRNA degradation of copper‐containing proteins such as Cu/Zn‐superoxide dismutases (SODs*),* plantacyanin and laccase‐like multicopper oxidases (Abdel‐Ghany & Pilon, 2008; Pilon, 2017; Shahbaz & Pilon, 2019). In *A. thaliana*, copper homeostasis is controlled by a conserved transcription factor, SPL7 (Squamosa Promoter Binding Protein–like7), and a recently discovered transcription factor, CITF1 (Copper Deficiency Induced Transcription Factor 1) (Bernal et al., 2012; Kropat et al., 2005; Yamasaki et al., 2009; Yan et al., 2017). SPL7 and CITF1 function in a complex integrated pathway that is essential for copper uptake, internal transport and delivery to reproductive organs (Yan et al., 2017).

In addition to mineral nutrient status, other factors that determine the plant reproductive success is the timing of the transition from the vegetative to the reproductive stage and the transition to senescence. Both developmental transitions depend on diverse endogenous and environmental cues (Amasino, 2010; Cho et al., 2017; Johansson & Staiger, 2014; Koyama, 2018). The endogenous and exogenous cues that mediate the transition to flowering include age, hormones, photoperiod, temperature, and nutrient availability, and are generally classified into five pathways: photoperiod, vernalization, age, gibberellin, and autonomous. These pathways are integrated by the florigen, Flowering Locus T (FT) that is produced in the leaf and transported *via* the phloem to the shoot apical meristem to trigger the formation of flowers (Teotia & Tang, 2015). Studies in *A. thaliana* have shown that the expression of *FT* is induced in leaves by day length/light, sucrose and its metabolite trehalose‐6‐phosphate (Cho et al., 2017, 2018; Möller‐Steinbach et al., 2010; Srikanth & Schmid, 2011; Wahl et al., 2013). Apart from them, *FT* expression is also regulated by two microRNAs, *miR156*, and *miR172* (Cho et al., 2017, 2018; Möller‐Steinbach et al., 2010; Teotia & Tang, 2015; Wahl et al., 2013; Wang et al., 2009). Our knowledge about the relationship between flowering time and copper availability is limited despite the important role of copper in plant growth and development.

Another important developmental transition that determines reproductive success is senescence. Natural leaf senescence ensures the remobilization of nutrients including minerals from senescing tissues to developing reproductive organs and seeds (Himelblau & Amasino, 2001; Leopold, 1961; Woo et al., 2019). Natural leaf senescence is typically triggered by the leaf age (Koyama, 2018; Woo et al., 2019). Environmental stresses, including nutrient deficiency, and hormones such as jasmonic acid (JA) are known to cause premature senescence (Leopold, 1961; Woo et al., 2019; Xie et al., 2014). While natural senescence increases reproductive success, premature senescence is often correlated with decreased yields. We have shown recently that JA levels increase in leaves of copper‐deficient *A. thaliana* suggesting that deficiency for this mineral causes premature senescence and could be among the reasons for the dropped seed yield (Yan et al., 2017). Hill et al. (1978) have shown, however, that copper deficiency delays chlorophyll degradation of mature wheat leaves and concluded that unlike other mineral deficiencies that trigger senescence, a copper deficiency might, in fact, delay it. Thus, whether copper deficiency causes premature senescence or delays it, is unclear. It is noteworthy that the expression of a copper chaperone *CCH* (*ATX1‐Like Copper Chaperone*) and a copper‐transporting ATPase, *RAN1* (*Responsive‐to‐antagonist1*) is upregulated by natural senescence in *A. thaliana* pointing to the important role of copper remobilization for the reproductive success (Himelblau, 2000; Himelblau & Amasino, 2001; Himelblau et al., 1998).

Here, we used *A. thaliana* (*cv*. Col‐0) to perform a systematic analysis of the effect of copper deficiency on key developmental processes including flowering and senescence that determine the reproductive success, substantiated the role of copper in male fertility and uncovered the role of copper in shoot architecture and stigma morphology. We have also used *A. thaliana* (*cv*. Ler) to test the reproducibility of some of copper deficiency‐caused phenotypes in other accessions.

## 2 MATERIALS AND METHODS

### 2.1 Plant materials and growth conditions

*Arabidopsis thaliana* (*cv*. Col‐0) was used in all experiments unless otherwise stated. Plants were grown hydroponically in magenta boxes to control copper concentrations as described in (Cho et al., 2017; Simpson & Dean, 2002; Yan et al., 2017). Copper was added at the indicated concentrations to the hydroponic solution as CuSO_4_. The standard (control) solution contained 250 nM CuSO_4_. Plants were grown in a growth chamber at 22°C constant temperature, 14‐hr light/10‐hr dark photoperiod at a photon flux density of 110 µmol/m^2^ s^−1^.

### 2.2 Phenotyping and seed set analysis

All measurements including flowering time, rosette leaf length and number, and primary inflorescence height were performed upon transition to flowering, i.e., appearance of the first open flower. For the rosette leaf length measurements, three leaves, either from the third or fourth bottommost, were measured for each plant, and then the average length was reported. Analyses of the number of floral buds and their fresh weight were performed five days after plants transitioned to flowering. For the floral buds fresh weight, the three outermost buds were chosen for measurements and the average weight was reported. Number of primary inflorescences and axillary branches were evaluated upon maturation of the primary inflorescence in each plant, i.e., appearance of the yellowish siliques (for plants grown under control condition or 250 nM copper) or drying out of the primary inflorescence (for copper‐deficient plants).

Seed number was analyzed upon maturation of the primary inflorescence. In brief, five to ten siliques were collected from primary inflorescences of five to ten independent mature plants and chlorophyll was removed by incubating siliques in 70% ethanol for several days. The number of seeds per silique was counted manually by dissecting each silique. All measurements were done using plants from at least three independent experiments with five to ten individually grown plants analyzed in each experiment.

### 2.3 Copper and auxin rescue treatment

Arabidopsis wild‐type plants were grown hydroponically without CuSO_4_.To test the role of copper and IAA on shoot branching, after three weeks of growth, rosette leaves and apical meristems of a subset of plants were sprayed for five consecutive days with either 1 µM CuSO_4_ or 1 µM IAA. In analyzing the effect of copper on the seed set, plants were continued to be sprayed with 1 µM CuSO_4_ once per week until maturation, i.e., formation of siliques. A mock treatment was performed using the deionized water for both rescue experiments.

### 2.4 Pollen germination and viability assays

Pollen grains were isolated from anthers at the stage 13–14 of flower development (Sanders et al., 1999). For the analysis of the pollen grain number, 10–30 individual flowers from at least 10–30 independently grown plants were used and pollen was manually released from anthers on a media containing 0.7% (w/v) agar spread onto a microscope slide. Counting was performed after collecting images using the Axio Imager M2 microscope (Zeiss).

Pollen viability was evaluated using fluorescein diacetate (FDA) according to (Bou Daher et al., 2009). Briefly, a 10 mg/ml FDA stock was prepared in acetone and stored at −20°C. A working solution of FDA was made by diluting FDA stock in 10% sucrose solution to a final concentration of 0.2 mg/ml. Pollen grains were released from five to ten open flowers by tapping anthers into 200 µl FDA solution followed by 5 min incubation in the dark. An aliquot (50 µl) was then transferred to a microscope slide and viable pollen was analyzed by fluorescence microscopy using the Axio Imager M2 microscope (Zeiss) and the FITC filter set.

Pollen germination was analyzed as described in (Fan et al., 2001). Briefly, pollen grains were spread on pollen germination media for overnight growth at 25°C. After 24 hr of incubation, the germinated pollen grains were imaged using the Axio Imager M2 microscope (Zeiss) and counted using ImageJ software. For the copper rescue experiment, CuSO_4_ was added directly to the cooled pollen germination media (50°C) to a final concentration of 20 nM. For the L‐ascorbate rescue experiment, L‐ascorbate was added directly to the cooled pollen germination media (50°C) to a final concentration of 5 µM.

### 2.5 Analysis of anther dehiscence

Anther dehiscence was analyzed according to (Yan et al., 2017). In brief, anthers from stage 14 of flower development were analyzed using a Leica S6E stereomicroscope at 40X magnification. For plants grown without CuSO_4_, 50 flowers from ten independently grown plants were analyzed. For the control condition, 20 flowers from 10 independently grown plants were analyzed.

### 2.6 Preparation of ultra‐thin sections from flowers

The effect of copper deficiency on anther morphogenesis was analysed by light microscopy on ultra‐thin sections using the procedure modified from (Zhao et al., 2002). Briefly, the entire primary inflorescences containing floral buds and flowers at different developmental stages were fixed for three hours in 2% glutaraldehyde (v/v) in 0.05 M cacodylate buffer (pH 7.4). After washing three times, 10 min each time in cacodylate buffer, samples were dehydrated for 10 min in ethanol series of 25%, 50%, 70%, 85%, and two times in 100%. The molecular sieve was used in 100% ethanol to trap any extra water that may have been accumulated in ethanol. Samples were then placed in 100% absolute acetone with molecular sieve for two more changes, 10 min each. All the above‐mentioned steps were conducted on ice. Inflorescences were dissected into individual buds and flowers prior to transferring to epoxy resin (Quetol 651, catalogue # 14640 VWR) series. Individual buds/flowers were then embedded in epoxy: acetone (1:3 ratio) for four to eight hours, transferred to epoxy: acetone (1:1 ratio) for four to eight hours, transferred to epoxy: acetone (3:1 ration) for eight hours, and 100% epoxy for 12 hr (or overnight). Finally, dissected samples were polymerized in molds at 60°C for 12 hr. Ultra‐thin cross sections (1 µm) were obtained using an ultramicrotome (Leica‐Ultracut‐UCT) equipped with a diamond knife. Sections were then heat fixed on glass slides for about 15 min. Obtained sections were stained with 0.5% toluidine blue (Sigma‐Aldrich) for one minute and rinsed three times with deionized water. Images were taken using Axio Imager M2 microscope (Zeiss).

### 2.7 Lignin staining

Anthers were dissected from flowers at stage 13 to 14 of flower development (Sanders et al., 1999) and stained with phloroglucinol‐HCl (Hao et al., 2014b). The staining solution was prepared by mixing two parts of 2% (w/v) phloroglucinol in 95% (v/v) ethanol with one part of concentrated HCl. For visualizing lignin in the inflorescence stem, transverse hand sections of stems were fixed in a solution comprised of three parts absolute ethanol to one part acetic acid for 15 min, then rinsed with deionized water and stained with phloroglucinol‐HCl. Samples were imaged 10 min after staining using the Axio Imager M2 microscope (Zeiss, Inc). Images were collected with the high‐resolution AxioCam MR Camera and processed using the Adobe Photoshop software package, version 12.0.

### 2.8 ROS staining of pollen grains using CM‐H2DCFDA

ROS accumulation in pollen grains was evaluated using the general ROS sensor 5‐(and 6)‐chroromethyl‐2′,7′‐dichlorodihydrofluorescein diacetate (CM‐H2DCFDA) that is converted to the highly fluorescent 2′,7′‐dichlorofluorescein (DCF) upon oxidation by ROS (Chen et al., 2010). The procedure was based on (Muhlemann et al., 2018). In brief, mature pollen grains from plants grown hydroponically with or without 250 nM CuSO_4_ were released into the pollen germination media (Fan et al., 2001) with 5 µM CM‐H2DCFDA (Thermo Fisher). Pollen grains were then incubated at 28°C for 20 min and pelleted by a quick spin. The staining solution was then replaced with pollen germination media followed by the DCF fluorescence visualization using the FITC filter set on the Axio Imager M2 microscope. The background fluorescence levels were determined for each sample using unstained pollen grains. Pollen grains with fluorescence above the background levels were counted.

### 2.9 Cytochrome *c* oxidase enzyme activity

Plants were grown hydroponically with or without 250 nM CuSO_4_ for four weeks (in case of rosette leaves) or seven to eight weeks (in case of floral buds). Mitochondria were extracted according to the procedure described in (Keech et al., 2005). This experiment was done three independent times for each tissue, each with three independent biological replicates, with all experiments showing similar results. Cytochrome *c* oxidase activity was measured using Cytochrome *c* Oxidase Assay Kit (Sigma‐Aldrich, catalog number CYTOCOX1) according to the manufacturer's instructions.

### 2.10 Isolation of total RNA and RT‐qPCR

Tissues were collected from plants grown hydroponically at the indicated copper concentrations, flash‐frozen in liquid nitrogen, and stored at –80°C prior to analyses. All samples were harvested between seven and eight Zeitgeber time, unless otherwise stated. Total RNA was isolated using TRIzol reagent (Invitrogen) according to the manufacturer's instructions. One microgram of total RNA was then treated with DNase I (New England Biolabs) prior to the first‐strand cDNA synthesis using AffinityScript RT‐qPCR cDNA synthesis kit (Agilent Technologies). RT‐qPCR analysis was conducted using iQ SYBRGreen Supermix (Bio‐Rad) according to manufacturer's instructions in the CFX96 real‐time PCR system (Bio‐Rad). *AtACT2* (AT3g18780) was used as a reference gene for data normalization. RT‐qPCR experiments were conducted using three independent experiments, each with three technical replicates. The list of oligos is shown in Table S1.

## 3 RESULTS

### 3.1 Copper deficiency increases shoot branching that can be partially rescued by auxin and fully by copper

**FIGURE 1:**
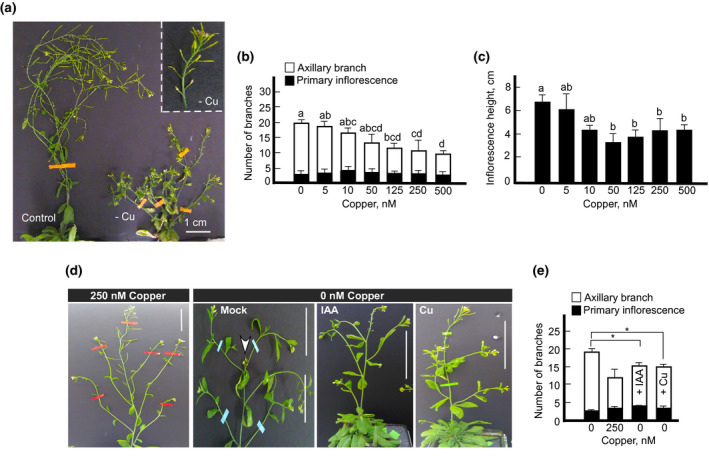
Copper deficiency alters shoot architecture in Arabidopsis. (a) Shows representative images of plants grown hydroponically with or without 250 nM CuSO_4_ until the reproductive stage. Inset shows a close‐up of an infertile copper‐deficient plant. (b) Plants were grown hydroponically under indicated copper concentrations. The number of primary inflorescences (black bars) and axillary branches (white bars) was evaluated at the late reproductive stage (stage 8 of *A. thaliana* development). (c) shows the height of a primary inflorescence upon transition to flowering (upon opening of the first flower on primary inflorescence) in plants grown under different copper concentrations. In (b) and (c) values are mean ± SE (*n* = 3 independent experiments with 5–10 plants analyzed in each experiment). Levels not connected by the same letter are significantly different (*p* < .05, Tukey‐Kramer HSD test using JMPPro 14). (d) Shows representative images of plants grown hydroponically with or without 250 nM CuSO_4_. Deionized water (Mock), 1 µM auxin (IAA) or 1 µM CuSO_4_ (Cu) were applied on rosette leaves and apical meristem for five days. Then, plants were sprayed once per week with copper until silique formation. A white arrow points to a partially aborted apical flower on the primary inflorescence. Experiments were done at least three independent times, with three to five individually grown plants used in each experiment. Scale bar = 1 cm in (a) and (d). (e) Quantification of primary inflorescences (black bars) and axillary branches (white bars) for plants shown in part (d). Asterisk (*) indicates statistically significant differences versus copper‐deficient plants (*p* < .05)

We first tested the effect of different copper concentrations on the growth and development of *A. thaliana* because this has not been done comprehensively. We grew plants hydroponically in different CuSO_4_ concentrations ranging from zero (no copper added) to 500 nM. As would be expected due to the essential nature of copper, plants grown under low copper developed chlorotic spots on rosette leaves upon transition to flowering (Figure S1a) and had an overall smaller stature and smaller rosette leaves compared to plants grown under control conditions (i.e., 250 nM CuSO_4_) (Figure S1). Unexpectedly, plants grown under copper deficiency developed more axillary branches (Figure 1a,b) and had a longer primary inflorescence upon transition to flowering compared to plants grown under control conditions (Figure 1c). We also noted that the apical flower bud on the primary inflorescence was aborted in plants grown under copper deficiency (Figure 1d‐I,II). This suggested that the increased branching might be related to the removal of auxin‐dependent apical dominance. Consistent with this suggestion, exogenous application of indole‐3‐acetic acid (IAA) to the shoot apex decreased shoot branching (Figure 1d‐III,E). Exogenous application of copper to the shoot apex also rescued shoot branching defect of copper‐deficient plants (Figure 1d‐IV,E). It is noteworthy that copper but not IAA application almost fully restored the overall plant size. These data link copper to auxin signaling in establishing inflorescence architecture. The concentration of 250 nM CuSO_4_ was used as a control for comparisons from here on.

### 3.2 Copper deficiency delays transition to flowering, reduces the number of flowers and alters the expression of *FT* and *miR172*

**FIGURE 2:**
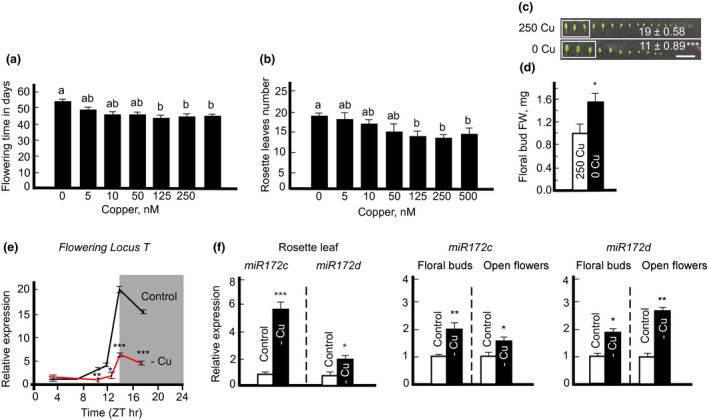
Copper deficiency delays transition to flowering by altering the expression of *FT* and *miR172* in Arabidopsis. Plants were grown hydroponically with the indicated copper concentrations in (a) to (c) or without (‐ Cu) or with 250 nM CuSO_4_ in (d) to (f). (a) and (b) show flowering time and the number of rosette leaves, respectively, upon transition to flowering (*i.e.,* the appearance of first open flower). Presented values are arithmetic means ± SE (*n* = 3 independent experiments with 5–10 plants analyzed in each experiment). Levels not connected by the same letter are significantly different (*p* < .05, based on Tukey‐Kramer HSD test). (c) and (d) show the number and fresh weight, respectively, of floral buds five days after the transition to flowering. Presented values are arithmetic means ± SE. Asterisks (*and ***) indicate statistically significant differences versus controls (*p* < .05, and < 0.0001, respectively, determined with Student's *t* test). (e) Diurnal time course expression pattern of *Flowering Locus T* (*FT*) in four‐week‐old rosette leaves. Samples were collected based on Zeitgeber time, where the Zeitgeber hour one is the first hour of light after the dark period. (f) Transcript abundances of *miR172c* and *d* in four‐week‐old rosette leaves and flowers. For analysis using rosette leaves, plants were grown hydroponically with 250 nM CuSO_4_ for four weeks and then transferred to a fresh medium lacking copper and grown for one more week to induce copper deficiency. Values shown are arithmetic means ± SE (*n* = 3 independent experiments with at least 3 to 5 plants analyzed in each experiment). Asterisks, * (*p* < .05), ** (*p* < .01) and *** (*p* < .001), indicate statistically significant differences compared to control condition as determined with Student's *t* test

It has been shown previously that *Chrysanthemum morifolium* grown under copper deficiency flowers later compared to copper‐sufficient plants (Graves & Sutcliffe, 1974). We noticed that copper‐deficient *A. thaliana* flowers later too. Here, we tested whether the late flowering under copper deficiency is caused by the delayed vegetative‐to‐reproductive stage transition or the slower growth rates of plants. The time to flowering and the rosette leaf number are used as common indicators for monitoring the time from the vegetative‐to‐reproductive stage transition in *A. thaliana* (Pouteau & Albertini, 2009). We found that copper deficiency not only delayed the time to flowering but also increased the number of rosette leaves upon transition to flowering and thus, delayed the transition from the vegetative to the reproductive stage (Figure 2a,b). By contrast, the number of floral buds on the primary inflorescence upon transition to flowering was significantly reduced in copper‐deficient plants (Figure 2c). Surprisingly, the copper‐deficient plants had larger and heavier floral buds (Figure 2c,d).

The transition to flowering is associated with the expression in the rosette leaves and transport to the shoot apical meristem of the floral activator, *Flowering locus T* (*FT*) and a floral identity marker, *miR172* (Duan et al., 2017; Díaz‐Manzano et al., 2018). We, thus, hypothesized that the delayed vegetative‐to‐reproductive stage transition in *A. thaliana* under copper deficiency can be caused by the decreased expression of *FT* and *miR172*. As we predicted, the transcript abundance of *FT* was significantly reduced at ZT10 (Zeitgeber time 10), ZT12, and ZT14 in leaves of plants grown without added copper compared to plants grown under control conditions. This reduction continued even through the dark cycle (Figure 2e).

We then tested *miR172* expression in both rosette leaves and flowers (Figure 2f). Among five isoforms tested, we could only detect isoforms *c* and *d*. We found that *miR172c* and *d* transcript abundances were significantly increased in both rosette leaves and flowers of copper‐deficient plants (Figure 2f). Together, these results suggest that copper deficiency‐driven delayed flowering is, in part, mediated by changes in the expression of *FT* and is independent of *miR172*.

### 3.3 Copper deficiency reduces fertility and impacts both reproductive organs

**FIGURE 3:**
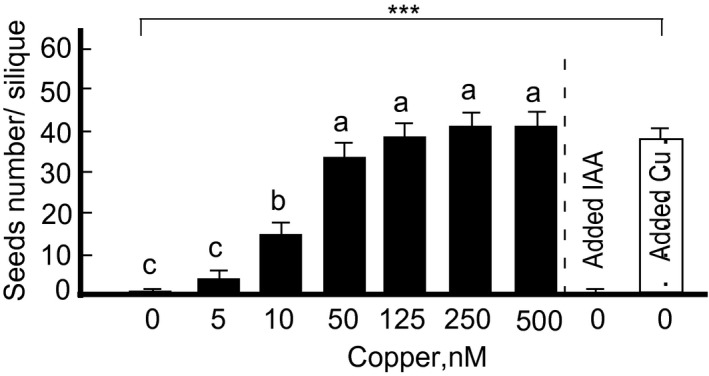
Copper‐deficiency caused infertility is rescued by external copper. Seeds number per silique are shown for plants grown under different copper concentrations as indicated. A dashed line separates results from the experiment in which the seed set was analyzed in copper‐deficient plants that were sprayed with either 1 µM copper or IAA as described in Figure 1. Note that only external copper rescued the poor seed set of copper‐deficient plants. Values are means ± SE (*n* = 3 independent experiments with 5–10 plants analyzed in each experiment). Levels not connected by the same letter are significantly different (*p* < .05, based on Tukey‐Kramer HSD test). In case of copper and IAA application, *** (*p* < .001 based on the Student's *t* test), indicates statistically significant differences compared to copper‐deficient plants

Our recent studies have shown that copper is associated with both anthers and pistils in *A. thaliana* (Yan et al., 2017). This finding suggested that copper is needed for the fertility of both reproductive organs. To test this prediction, fertility and seed production were evaluated using reciprocally crossed wild‐type *A. thaliana* grown with or without copper. At the onset of this study, we evaluated the effect of different concentrations of copper on seed production. As would be expected, plants were sterile when grown without copper supplementation (Figure 3). Increasing copper concentration in hydroponic medium improved fertility (Figure 3).

To find which reproductive organs were affected by copper deficiency, reciprocal crosses were conducted between control plants (grown in 250 nM CuSO_4_) and copper‐deficient plants. As shown in Table 1, the seed production was severely affected when gynoecium of plants grown under control conditions was fertilized with pollen from plants grown without copper. However, seed production was completely abolished when the gynoecium of a copper‐deficient plant was a recipient of pollen from a control plant (Table 1). Together, these results indicated that copper deficiency causes defects in both reproductive organs with the most pronounced effect on the gynoecium.

**Table 1:** Copper deficiency reduces fertility of both androecium and gynoecium in *A. thaliana*. Reciprocal crosses were conducted between plants grown with or without 250 nM CuSO_4_. Statistical significant levels were determined by the Pearson's Chi‐squared test (*x* ^2^) with a minimum threshold set at *p* < .05 compared to reciprocally crossed plants grown under control conditions

Cross	Seeds/silique Mean ± SE	Siliques analyzed	*p*‐value
Female_control × male_control	24 ± 2.02	3	1
Female_control × male_0 Cu	11 ± 3.13	8	<.01
Female_0 Cu × male_control	0	22	<.01

### 3.4 Copper deficiency reduces stigmatic papillae formation

**FIGURE 4:**
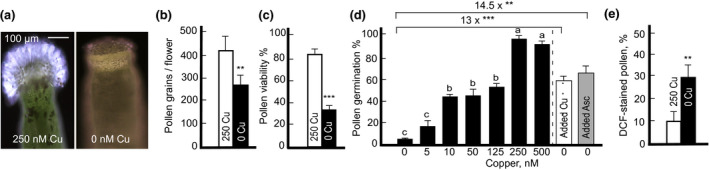
Copper deficiency dramatically reduces stigma and pollen grains fitness. (a) Shows representative light microscopy images of the stigma of plants grown hydroponically with or without 250 nM CuSO_4_. Note that stigma from a copper‐deficient plant is almost papilla‐less. The number of pollen grains per flower (b) and pollen viability (c) in plants grown with or without 250 nM CuSO_4_. (d) Shows results from the in vitro pollen germination assays of plants grown under indicated copper concentrations. Dashed vertical line is used to separate experiments were 20 nM copper or 5 µM L‐ascorbate were added directly to the pollen germination medium. (E) ROS accumulation in pollen grains as determined by the evaluation of DCF fluorescence levels. In (b) to (e) values are means ± SE (*n* = 3 independent experiments with 5–10 plants analyzed in each experiment). Asterisks (** and ***) indicate statistically significant differences compared to control condition in (B), (C), and (E) or copper‐deficient condition in (d) with *p* < .01 and 0.001 (Student's *t* test). Levels not connected by the same letter are significantly different (*p* < .05, based on Tukey‐Kramer HSD test)

To evaluate the role of copper in the gynoecium fertility, we took a closer look at pistils and noticed that the stigma in almost 90% of the copper‐deficient plants lacked or had shorter papillae (Figure 4a). Because stigmatic papillae serve as attachment sites for pollen and are required for fertilization (Kang et al., 2003; Thorsness et al., 1993), we speculate that papillae length reduction or complete abolishment under copper deficiency is a major contributing factor to the defect in female fertility (Table 1).

### 3.5 Copper deficiency reduces pollen number, viability and germination, and increases ROS production

Concerning the reduced male fertility (Table 1), copper‐deficient plants produced fewer pollen grains (Figure 4b), more than 60% of which were unviable compared to plants grown under control condition (Figure 4c). Decreasing copper concentration in the plant growth medium also decreased pollen germination and nearly abolished it when plants were grown without copper (Figure 4d and Figure S2a). The exogenous application of copper directly to the pollen germination medium increased pollen germination by 13 fold (Figure 4d and Figure S2b). Because copper deficiency causes oxidative stress, we asked whether observed defects could be associated with the accumulation of reactive oxygen species (ROS). In line with our expectation, the exogenous application of an antioxidant, L‐ascorbate, directly to the pollen germination medium increased pollen germination by 14.5 fold (Figure 4d and Figure S2b). To evaluate ROS level in pollen grains, we used the general ROS sensor, 5‐(and 6)‐chloromethyl‐2′,7′‐dichlorodihydrofluorescein diacetate (CM‐H2DCFDA) that is converted to the highly fluorescent 2′,7′‐dichlorofluorescein (DCF) upon oxidation by cellular ROS (Muhlemann et al., 2018). We found that pollen from copper‐deficient plants indeed accumulated more ROS compared to plants grown under control conditions (Figure 4e). Together, these results suggest that at least some of the pollen fertility defects of copper‐deficient plants, namely pollen germination, are due to copper deficiency‐promoted oxidative stress.

To identify other copper‐dependent contributors to pollen fertility, we examined the activity of cytochrome *c* oxidase (COX), a copper‐containing protein complex that plays a crucial role in the mitochondrial respiratory chain, and is essential for the cellular energy production (Droppa et al., 1984). Due to difficulty in collecting enough pollen material for the isolation of mitochondria, we measured COX activity in mitochondria that were isolated either from rosette leaves or floral buds of plants grown with or without 250 nM copper. As would be expected because of the essential role of copper in COX function, copper deficiency decreased COX activity by almost 3‐fold compared to that in the control condition in both tissues tested (Table 2). These results suggested that the observed fertility defects might be, in part, due to the decreased cellular energy production.

**Table 2:** Copper deficiency reduces cytochrome *c* oxidase activity in *A. thaliana* rosette leaves and floral buds. Plants were germinated and grown hydroponically with or without 250 nM CuSO_4_ for four weeks (in case of rosette leaves) or seven to eight weeks (in case of floral buds). Mitochondria were extracted according to Keech et al. (2005). This experiment was done three independent times for each tissue, each with three independent biological replicates, with all experiments showing similar results. A representative result of three experiments is shown for each tissue type as mean values ± *SD*. ***Indicates statistically significant difference compared to control condition with *p* < .0001, using the Student's *t* test

Tissue	Copper concentration	Cytochrome c oxidase (units/ml) Mean ± *SD*
Rosette leaves	250 nM	1,151 ± 30.5
	0 nM	361 ± 10.5***
Floral buds	250 nM	1,005 ± 26.2
	0 nM	312 ± 6.6***

### 3.6 Copper deficiency compromises anther and pistil specification

**FIGURE 5:**
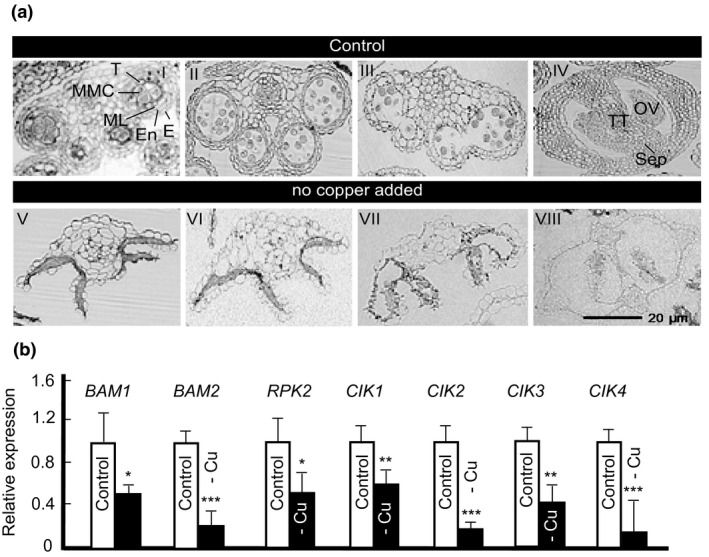
Copper deficiency causes malformation of both androecium and gynoecium during different developmental stages. (a) Representative images of the floral bud cross sections showing an anther and a pistil. Plants were grown hydroponically with or without 250 nM CuSO_4_. I, II, III, IV are for plants grown in control conditions. V, VI, VII, and VIII are for copper‐deficient plants. I and V are early stages of anther development, in which, microspore mother cells are present. II and VI are middle stages of anther development in which microspores are released. III and VII are late stages of anther development in which anthers are bilocular and contain tricellular pollen grains. IV and VIII are gynoecium cross sections present at the later stages of anther development (i.e., III and VII). E, epidermis; En, endothecium; ML, middle layer; MMC, microspore mother cells; OV, ovule; Sep, Septum; T, tapetum; TT, transmitting tract. (b) Transcript abundances of anther‐specific genes in floral buds. Plants were grown hydroponically with or without 250 nM CuSO_4_. Values are means ± SE (*n* = 3 independent experiments with at least 3 to 5 plants analyzed in each experiment). Asterisks, *, **, and *** indicate statistically significant differences compared to control condition with *p* < .05, 0.01, and 0.001, respectively, using Student's *t* test

To gain further insight into the effect of copper deficiency on reproductive organs, ultrathin transverse sections were prepared from *A. thaliana* floral buds collected at different developmental stages from plants grown with or without 250 nM CuSO_4_. The anther wall of plants grown under copper sufficient condition contained four defined cell layers including epidermis, endothecium, middle layer, and tapetum (Figure 5a‐I,‐II,‐III). However, the wall of anther lobes in copper‐deficient floral buds looked contorted without any defined cell layers in all developmental stages, and as early as at the stage of pollen mother cell formation (Figure 5a‐V). The same undefined structures remained throughout the middle and late stages of anther development in copper‐deficient plants (Figure 5a‐VI,‐VII). Similarly, a cross‐section through the gynoecium of the copper‐deficient flowers showed undefined structures (Figure 5a‐VIII) compared to that in the control condition (Figure 5a‐IV). Together, these observations indicate that copper deficiency adversely affects the morphology of both androecium and gynoecium in their development.

### 3.7 Copper deficiency decreases the expression of genes involved in early anther development

We then tested the effect of copper deficiency on the expression of genes associated with early stages of anther development. Specifically, we analyzed the expression of *BAM1* and *BAM2* (*Barely Any Meristem*), which encode CLAVATA1‐related leu‐rich repeat receptor‐like kinases (LRR‐RLKs) (Hord et al., 2006), *RPK2* which is also an LRR‐RLK (Mizuno et al., 2007), and *CIK1* to *CIK4* (*Clavata3 Insensitive Receptor Kinase1* to *4*) known as co‐receptors of BAM1, BAM2, and RPK2 (Cui et al., 2018). RT‐qPCR results showed that the transcript abundance of all marker genes was significantly reduced under copper deficiency compared to the control condition (Figure 5b). These results were consistent with the observed anther developmental defects of copper‐deficient plants (Figure 5a) and provided another layer of evidence that copper deficiency causes malformation of reproductive organs.

### 3.8 The expression of *copper‐microRNAs* is upregulated in flowers of copper‐deficient Arabidopsis

**FIGURE 6:**
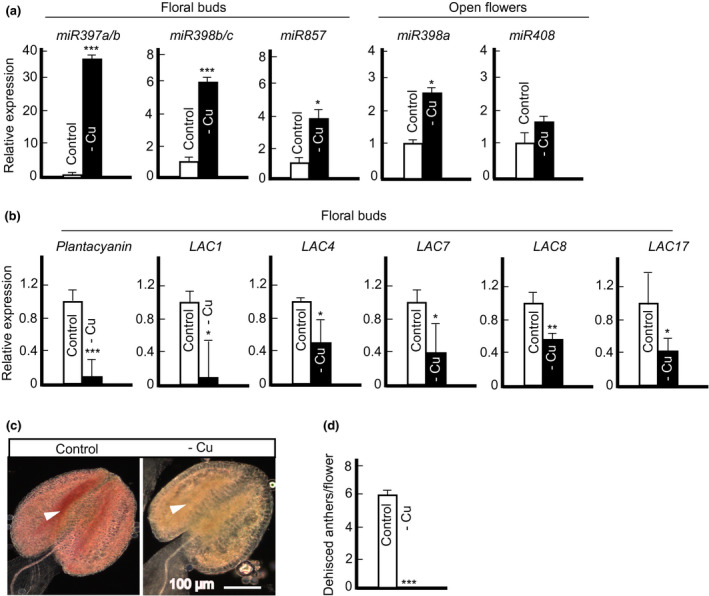
Copper deficiency upregulates the expression of copper miRNAs and reduces the expression of laccases and lignin accumulation. Transcript abundance of Cu‐miRNAs (a), *plantacyanin* and lignin biosynthesis genes, *laccases*, (B) in open flowers or floral buds under copper deficiency. In (a) and (b), values are means ± SE (*n* = 3 independent experiments with 3 to 5 plants analyzed in each experiment). Asterisks * (*p* < .05), ** (*p* < .001) and *** (*p* < .001) indicate statistically significant differences compared to control condition determined by Student's *t* test. (c) A representative image of the anther from plants grown with or without 250 nM copper. Phloroglucinol‐HCl stains lignin in red. White arrowheads indicate stomium region in the anther wall. Representative images are presented here from five independent experiments, each included at least 10 plants per condition. Note that copper‐deficient anther lacks lignin in the stomium region in addition to everywhere else. (d) Dehisced anthers per single flower. Values are means ± SE (*n* = 3 independent experiments). Thirty individual flowers from 10 individually grown plants without copper were analyzed. For control condition (250 nM CuSO_4_), 10 individual flowers from 5 individually grown plants were analyzed. Asterisks (***) indicate statistically significant difference compared to control condition with *p* < 0001 determined by Student's *t* test

Transcript abundance of several microRNAs including *miR397*, *miR398*, *miR408,* and *miR857* is increased by copper deficiency in roots and leaves of *A. thaliana* (Abdel‐Ghany & Pilon, 2008; Pilon, 2017). Here, we tested whether the expression of these *copper‐microRNAs* also increases under copper deficiency in *A. thaliana* flowers. To observe the dynamics and the specificity of their expression during flower development, we tested the expression of *copper‐miRNAs* in floral buds and open flowers. Among them, we detected the expression of *miR397a/b, miR398b/c* but not *miR398a,* and *miR857* (single gene) in floral buds but not in open flowers. On the other hand, we could detect *miR398a* and *miR408* (single gene) expression in open flowers but not in floral buds. RT‐qPCR results showed that the transcript abundance of *copper‐microRNAs* tested, except for *miR408*, was significantly increased under copper deficiency (Figure 6a), suggesting that they are involved in the response to copper deficiency in floral organs as well.

### 3.9 Copper deficiency reduces the expression of *laccases* in flowers of Arabidopsis

We next tested the effect of copper deficiency on the expression of some of the known copper‐microRNA targets including *laccases* (*LAC1*, *LAC4*, *LAC7*, *LAC8*, and *LAC17*) encoding multicopper oxidases that are implicated in lignin synthesis and *plantacyanin*, encoding a plant‐specific blue‐copper protein (Dong et al., 2005; Nersissian et al., 1998; Zhang, Zhang, et al., 2018; Zhao et al., 2013). We chose *LAC1, LAC4,* and *LAC17* based on their decreased expression in our previous RNA‐Seq data (Yan et al., 2017) in addition to *LAC7* and *LAC8* from (Abdel‐Ghany & Pilon, 2008). *miR397* targets *LAC4* and *LAC17,* while *miR408* and *miR857* target *plantacyanin* and *LAC7,* respectively. Although *LAC8* has no predicted *copper‐microRNA* target sites, its expression is regulated by the copper supply (Abdel‐Ghany & Pilon, 2008). We found that the transcript abundance of all *LAC* genes and *plantacyanin* was significantly decreased under copper deficiency in floral buds (Figure 6b). We also tested the effect of copper deficiency on *Cu‐microRNAs* and their corresponding targets in the primary inflorescence. The transcript abundance of all tested microRNAs and all tested *LAC* genes was significantly up‐ and down‐regulated, respectively, by copper deficiency in primary inflorescences (Figure S3).

### 3.10 Copper deficiency reduces lignin accumulation in anthers and reduces anther dehiscence

As the transcript abundance of several *LAC* genes including *LAC4* and *LAC17* was reduced in flowers under copper deficiency, we predicted that lignin deposition will be decreased in copper‐deficient plants as well. We found a dramatic reduction of lignin staining in anthers and primary inflorescence of copper‐deficient plants (Figure 6c and Figure S3c). Concerning anthers, lignin staining was observed in the stomium region of plants grown under control conditions while was nearly absent in this region in plants grown under copper deficiency (Figure 6c, white arrowhead). Lignin deposition was observed in the xylem, including the vessels, parenchyma, and the interfascicular region of inflorescence stems of plants grown under control condition. However, lignification was completely abolished in the interfascicular fibers and was only detectable in the xylem vessels in copper‐deficient plants (Figure S3c). Consistent with the important role of lignification in anther wall thickening and thus, anther dehiscence (Mitsuda et al., 2005), nearly 100% of anthers from copper‐deficient plants were indehiscent (Figure 6d).

### 3.11 The expression of senescence‐associated genes, *SAG12*, *SAG13,* and *WRKY53* is altered in young and mature leaves in copper‐deficient Arabidopsis

**FIGURE 7:**
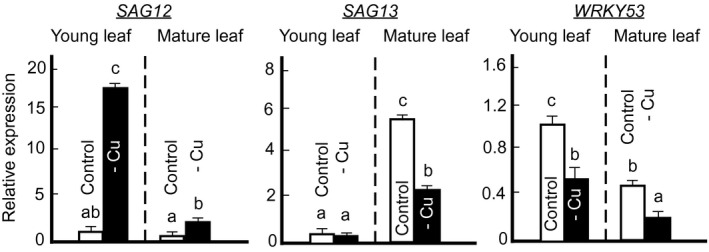
Copper deficiency decreases the expression of *SAG13* and *WRKY53* but triggers the expression of *SAG12* upon transition to bolting. Expression of *SAG12*, *SAG13*, and WRKY53, as senescence marker genes, in young and fully mature green rosette leaves upon bolting. Plants were grown hydroponically with or without 250 nM CuSO_4_ until bolting. The approximate plant age was five to six weeks. Shown are mean values ± *SD* of a representative experiment of three independent experiments. Each experiment analyzed three to five plants. Levels not connected by the same letter are significantly different (*p* < .05, based on Tukey‐Kramer HSD test). Data were normalized to the transcript abundance of corresponding genes in young leaves of plants grown under control conditions

Our recent studies in *A. thaliana* have shown that copper deficiency triggers the foliar accumulation of jasmonic acid (JA) (Yan et al., 2017). Because JA, among its other physiological functions, is also considered as one of the early signals of leaf senescence, we hypothesized that copper deficiency may trigger leaf senescence as well. To test our hypothesis, we evaluated the effect of copper deficiency on the expression of the senescence‐associated genes that are the downstream of JA targets, *SAG12*, *SAG13,* and *WRKY53* (Woo et al., 2019). As young leaves are more susceptible to copper deficiency than mature leaves due to poor copper mobility in the phloem (Broadley et al., 2012), we anticipated that young leaves might display more dramatic molecular responses of senescence. Consistent with our hypothesis, we found that the expression of *SAG12* was upregulated by nearly 2‐ and 17‐fold in mature and young leaves, respectively, under copper deficiency (Figure 7). Surprisingly, we found that the expression of *SAG13* and *WRKY53* was significantly downregulated in mature leaves of copper‐deficient versus copper‐sufficient plants (Figure 7). The expression of *WRKY53* was also significantly downregulated in young leaves of copper deficient versus copper‐sufficient plants (Figure 7). These data show that copper deficiency mounts a distinct transcriptional response of senescence‐associated genes, and perhaps, triggers distinct aspects of senescence.

### 3.12 Copper deficiency response in *A. thaliana* accession *Landsberg erecta* (*Ler*) is similar to *A. thaliana Col‐0*

To test whether copper‐deficiency responses of *A. thaliana* (*cv. Col‐0*) are also common in other popular laboratory accessions, we tested *A. thaliana* accession *Landsberg erecta* (*Ler*). *L*er is one of the most popular strains of *A. thaliana* after the reference ecotype Columbia (Col‐0) and is used widely for functional genomics studies (Torii et al., 1996; Zapata et al., 2016). As *Ler* harbors a mutation in the *ERECTA* gene (known as *er* mutation) that alters the inflorescence architecture (Zapata et al., 2016), here we only tested the effect of copper deficiency on flowering time and reproduction. Similar to *A. thaliana* (*cv. Col‐0*), *A. thaliana* (*cv. Ler*) grown without copper supplementation displayed chlorotic spots in rosette leaves, significantly smaller stature with smaller rosette size and late‐flowering phenotype (Figure S4a–c). We note, that unlike *A. thaliana* (*cv*. Col‐0), the primary inflorescence of copper‐deficient *cv*. Ler was significantly shorter than under control conditions and overall, the effect of copper deficiency was even more acute (Figure S4a). The reduced fertility of *Ler* plants under copper deficiency was also associated with abnormal papillae development and the reduced anther lignification (Figure S4e,f). While our results indicate that there are multiple common responses to copper deficiency between the two accessions, there are also the cultivar‐specific responses in *A. thaliana (cv. Ler*) and, perhaps, other accessions.

## 4 DISCUSSION

The important role of the micronutrient copper in plant fertility has been recognized for more than 30 decades. A range of crop species, including wheat, oat, barley, sweetcorn, and sunflower was used to show that copper deficiency affects reproduction more strongly than vegetative growth, may delay flowering and leads to male sterility (Dell, 1981; Graham, 1978; Graves & Sutcliffe, 1974). These pioneering studies have been very informative but yet scattered and raised more mechanistic questions including what are the sites of copper accumulation in flowers, which aspects of copper metabolic functions are important for ensuring successful fertility, which transport pathways are responsible for copper delivery to reproductive organs and how these transport pathways are regulated. In addition, a systematic and comprehensive analysis of the effect of copper deficiency on plant fitness with a focus on reproduction has not been yet conducted. Here, we used a model dicot *A. thaliana* (*cv*. Col‐0 and *cv*. Ler) to initiate a comprehensive analysis of molecular and mechanistic reasons underlying the copper‐deficiency mediated late flowering, poor pollen germination, and compromised reproduction.

### 4.1 Copper deficiency alters shoot architecture in Arabidopsis

While establishing the range of copper concentrations that can be regarded as adequate for the growth of *A. thaliana* in hydroponics, we noted that plants grown without copper supplementation had small rosette size (Figure S1). This finding was expected because of the essential role of copper in respiration and photosynthesis. In fact, about 50% of copper found in plants is present in chloroplasts, where it is bound to plastocyanin, a copper‐containing protein that mediates electron transfer between PSII and PSI (Weigel et al., 2003). Therefore, copper‐deficient plants have low rates of photosynthesis and reduced carbohydrate production that, in turn, is reflected in the reduced plant growth and development (Broadley et al., 2012; Brown & Clark, 1977; Ravet & Pilon, 2013). In addition, the reduced respiration‐based energy supply for the energy‐dependent processes are among the contributing factors for the decline of growth rates and failure to reach the full‐size potential of the developing leaf. With that notion, it was surprising to find that copper‐deficient plants had longer primary inflorescence upon transition to flowering and developed more axillary branches (Figure 1a–c). We also found that although copper‐deficient plants have significantly fewer floral buds (Figure 2c), the flower buds were larger and heavier than those in plants grown under control conditions (Figure 2d).

It is recognized that the plasticity of shoot architecture depends on differential activation of axillary buds, environmental conditions and interactions between systemically moving phytohormones auxin, strigolactones, and cytokinins (reviewed in (Teichmann & Muhr, 2015), (Wang et al., 2018) and (Domagalska & Leyser, 2011)). Among these phytohormones, the prominent role of auxin in exerting apical dominance is well‐established. Removal of the shoot apex (decapitation) results in lateral bud activation and shoot branching, while reapplication of exogenous auxin to the stump restores branching inhibition (reviewed in (Domagalska & Leyser, 2011). In our experiments, we noted that copper deficiency caused the abortion of the apical flower bud (Figure 1d). Thus, we speculated that copper deficiency promotes shoot branching *via* mitigating the apical dominance effect and altering auxin signaling. Consistent with this suggestion, the reapplication of IAA or copper to the shoot apex rescued the defect in the shoot apical dominance and decreased branching of copper‐deficient plants (Figure 1d,e). The relationship between copper and auxin homeostasis remains to be elucidated but it is noteworthy that copper promotes auxin accumulation and cell proliferation in the copper moss *Scopelophila cataracta* (Nomura et al., 2015), while excess copper prevents auxin redistribution in the root through interacting with an auxin efflux carrier, Pinformed1 (PIN1) (Yuan et al., 2013).

### 4.2 Copper deficiency delays vegetative to reproductive stage transition by reducing the expression of *FT* gene

To ensure successful reproduction, plants control their flowering time by changing their growth rates and/or altering the vegetative‐to‐reproductive stage transition (Cho et al., 2017; Schmalenbach et al., 2014; Simpson & Dean, 2002). Although it is accepted that poor nutrition tends to promote flowering, low phosphorus and nitrogen have distinct effects on flowering time in *A. thaliana* (Cho et al., 2017). Specifically, nitrate‐limiting conditions promote flowering independently of light, gibberellins, and autonomous pathways (Castro Marín et al., 2011). By contrast, phosphorus deficiency delays flowering (Kant et al., 2011). Our observations presented here and in Sheng et al. (2019) show that copper deficiency delays flowering in both *A. thaliana* ecotypes (Figure 2a and Figure S4c) and *B. distachyon*. It is unclear, however, whether delayed flowering under copper deficiency is a result of slower growth rates due to the reduced photosynthesis and respiration and/or a delayed developmental transition from the vegetative‐to‐reproductive stage. Our finding that copper deficiency has led to the accumulation of rosette leaves in *A. thaliana* (Col‐0) (Figure 2b) suggests the role of copper in developmental transition from the vegetative‐to‐reproductive stage. Interestingly, although the flowering time was delayed in copper‐deficient *A. thaliana* (*cv*. Ler) the number of rosette leaves did not change compared to plants grown under control conditions (Figure S4c,d) suggesting the ecotype‐specific responses to copper deficiency.

**FIGURE 8:**
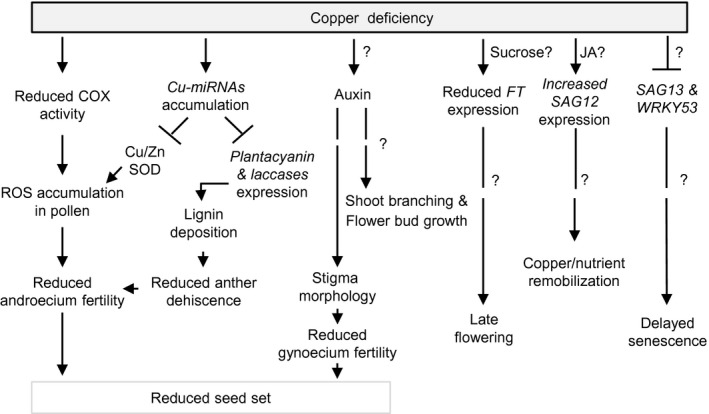
The proposed model of the effect of copper deficiency on development and reproduction of *Arabidopsis thaliana*. Copper deficiency causes multiple defects in *A. thaliana* including a significantly reduced seed set, altered shoot architecture, delayed flowering and senescence. These defects in copper‐deficient plants are caused by a variety of molecular adjustments that alter developmental events. Specifically, the reduced cytochrome *c* oxidase (COX) activity in leaves and flower buds together with the loss of Cu/Zn SODs, known copper miRNAs targets, contribute to ROS accumulation in pollen grains of copper‐deficient plants. The reduced COX activity also impacts the cellular energy level that is required for the pollen tube growth and successful fertilization. The loss of copper miRNA‐targeted laccases impacts lignification and contributes to anther indehiscence. Together, these events reduce male fertility of copper‐deficient plants. Copper deficiency also changes shoot branching and stigma morphology possibly *via* changes in the auxin level. Loss of the stigma papillae might contribute to female infertility under copper deficiency. Further, copper deficiency reduces the expression of the *FT* gene, causing the delay in the transition to flowering. Sucrose is a possible contributor, acting upstream of *FT*. Copper deficiency also triggers the accumulation of *SAG12* transcript, possibly through jasmonic acid (JA), suggesting its involvement in the copper/nutrient remobilization. On the other hand, the reduced expression of *SAG13* and *WRKY53* under copper deficiency suggests the overall delay in developmental senescence. Question marks (?) indicate components of the pathway not yet experimentally characterized

Although the specific role of copper in flowering time is yet to be determined, it is possible that plants stay longer in the vegetative stage to accumulate the critical level of photosynthates. In line with this suggestion are past studies showing that photosynthetic activity influences flowering (Bernier et al., 1993) and that *A. thaliana* exposed to strong irradiation flowers sooner, and has the increased levels of endogenous sucrose in leaves (King et al., 2008). Sucrose also promotes flowering in several species, and the exogenous application of a low concentration of sucrose partially rescues the late‐flowering phenotypes of Arabidopsis mutants (Bernier et al., 1993; Cho et al., 2018; Ohto et al., 2001). Trehalose‐6‐phosphate has been implicated in the regulation of flowering time in *A. thaliana* and the downregulation of trehalose‐6‐phosphate synthase expression significantly delays flowering even though the basal sucrose level remains unchanged (Cho et al., 2018; Wahl et al., 2013). It is suggested that sucrose functions in the leaf phloem while trehalose‐6‐phosphate functions in the shoot apical meristem to enhance the generation of florigens such as *FT* (Cho et al., 2018; Wahl et al., 2013). Specifically, the increased endogenous sucrose levels due to higher photosynthetic activity lead to higher expression of *FT*, hence, sucrose‐mediated signals are regarded to function upstream of *FT* and are intimately related to the plant photosynthetic capacity (King et al., 2008; Seo et al., 2011). In this regard, it is noteworthy that the transcript abundance of *FT* was significantly decreased in leaves of *A. thaliana* under copper deficiency (Figure 2e). Thus, it is possible that copper deficiency delays flowering time indirectly *via* reducing photosynthetic rates, decreasing the sucrose and perhaps, trehalose‐6‐phosphate level, which in turn, leads to the reduction of *FT* expression (Figure 8).

### 4.3 Copper deficiency increases the expression of *miR172* in leaves of *A. thaliana*

Among factors mediating the transition to flowering in *A. thaliana* is a conserved regulatory module including two microRNA families, miR156 and miR172, and their corresponding downstream targets. The transition to flowering is associated with the reduction of *miR156* accumulation and a concomitant increase in *miR172* and *FT* (Teotia & Tang, 2015; Wang et al., 2009). Since we found that the transcript abundance of *FT* is reduced in leaves of copper‐deficient plants (Figure 2e), we anticipated that the expression of *miR172* will be reduced as well. Unexpectedly, the transcript abundance of both *miR172c* and *d* isoforms that we were able to detect in leaves of *A. thaliana*, increased significantly under copper deficiency (Figure 2f). Why the upregulation of *miR172* did not lead to the upregulation of *FT* and concomitant transition to flowering, is not clear. It is possible that the miR172‐stimulated *FT* expression is overridden by severe copper deficiency that, in turn, is expected to reduce the photosynthetic capacity, sucrose accumulation and *FT* expression (Figure 8). It is also possible that the copper deficiency‐mediated increase of *miR172* transcript is not sufficient to promote floral induction under copper deficiency. Alternatively, copper deficiency may also activate floral repressors including *Flowering Locus C* (*FLC*) (Crevillen & Dean, 2011; Ortuño‐Miquel et al., 2019), which, in turn, will reduce *FT* expression and delay flowering time independently of *miR172*. Future studies will establish the interactions between copper deficiency and pathways controlling flowering time.

### 4.4 Copper deficiency reduces fertility and causes defects in both reproductive organs

We found that seed production improves in plants in a copper‐dependent manner and plants are nearly sterile when grown without copper supplementation (Figure 3). We also showed that foliar application of copper but not IAA rescues poor seed set of copper‐deficient plants (Figure 3). Copper deficiency‐mediated defects in reproduction have been linked to male infertility (Dell, 1981; Graham, 1978). Our recent x‐ray synchrotron‐fluorescence microscopy‐based analysis of the spatial distribution of copper in flowers of *A. thaliana* identified a bulk of copper in anthers (Yan et al., 2017). Failure to deliver copper to anthers in the mutant lacking two regulators of copper homeostasis, SPL7 and CITF1, results in a dramatic loss of fertility (Yan et al., 2017). However, Yan et al., 2017 have also shown that in addition to anthers, copper is associated with pistils, suggesting that this mineral can function in female fertility as well. Consistent with this suggestion results from reciprocally crossed *A. thaliana* with one parent grown under copper sufficiency while another under copper deficiency, implicated copper in the fitness of both male and female gametophytes (Table 1). Moreover, we found that the gynoecium is much more impacted by copper deficiency than androecium. This was evidenced by the failure of plants to produce seeds when the female of a copper‐deficient plant was a recipient of pollen from a copper‐sufficient plant. By contrast, plants, grown under copper sufficiency and fertilized with pollen from the copper‐deficient plant, still produced some seeds, albeit at a significantly reduced level (Table 1).

Furthermore, we noticed that stigmas of both *A. thaliana* ecotypes Col‐0 and Ler grown under copper deficiency had either reduced papillae formation or had severely reduced papillae length (Figure 4a and Figure S4f). Because metabolically active papillae cells are required for successful pollen tube growth (Kandasamy et al., 1993), the infertility of gynoecium of copper‐deficient plants might be, in part, caused by a defect in papillae formation. It is noteworthy that the defect in copper delivery to reproductive organs in the mutant lacking a copper transporter YSL3 also leads to aberrant stigma formation in *B. distachyon* (Sheng et al., 2019). Why copper deficiency causes defects in stigma morphology is unclear. It is noteworthy that high amounts of auxin have been associated with the stigma in *A. thaliana* (Aloni et al., 2006) and mounting evidence suggests that auxin plays an important role in apical–basal patterning that includes stigma and style patterning of the gynoecium. For example, the reduced development of the stigmatic papillae observed in *A. thalian*a mutants for the *bHLH* family member *SPATULA* (*SPT*) can be rescued by blocking auxin transport using naphthylphthalamic acid (NPA) or by inducing the expression of the *STYLISH1* (*STY1*) that activates the auxin biosynthesis gene *YUCCA4* (Nemhauser et al., 2000; Sohlberg et al., 2006) and reviewed in (Marsch‐Martínez & de Folter, 2016). Thus, it is tempting to speculate that copper deficiency‐caused defect in papillae formation is linked to auxin homeostasis. In addition, we also found that floral buds of copper deficient plants are larger and heavier compared to controls (Figure 2c,d). It is now well‐recognized that auxin controls flower formation and differentiation, and that free auxin highly accumulates not only in stigmas but also in anthers whereby suppressing the basipetal and acropetal development of neighboring flower tissues (Aloni et al., 2006; Zhao, 2018). It is possible that copper deficiency reduces auxin accumulation in stigma and anthers contributing to these organs abnormalities (Figures 4a and 5a and Figure S4f), which, in turn, leads to auxin accumulation in perianth tissues and floral buds growth.

Results presented here also substantiate the role of copper in male fertility. Specifically, we show that copper deficiency significantly reduced the number of pollen grains, pollen viability, and germination in *A. thaliana* (Figure 4b–d and Figure S2). These results are consistent with past findings showing that anther and pollen development are sensitive to copper status (Agarwala et al., 1980, Jewell et al., 1988, Pandey, 2010). Graham (1975) reported that male sterility of wheat occurs near the meiosis stage of pollen mother cells. Using cross‐pollination, this group has shown that the copper deficiency‐caused infertility is due to pollen sterility but not due to the female gametophyte. This contrasts with our findings in *A. thaliana*, in which, cross‐pollination results showed that female fertility is entirely reduced under copper deficiency (Table 1). This discrepancy may be due to the species‐specific sensitivity to copper deficiency.

The defect in pollen fertility was also observed in *A. thaliana*, *B. distachyon,* and *Oryza sativa* mutants lacking transporters that mediate copper delivery to reproductive organs (Chu et al., 2010; Sheng et al., 2019; Zhang, Lu, et al., 2018). We showed that adding copper directly to the pollen germination medium rescued pollen germination defect of copper‐deficient *A. thaliana* (Figure 4d and Figure S2). This is consistent with the previous study in rice, where copper application rescued pollen germination defect of the rice mutant lacking copper‐nicotianamine transporter, OsYSL16 (Zhang, Lu, et al., 2018). Here we also showed that copper deficiency‐caused defect in pollen germination is, in part, caused by the altered redox status of the pollen grains and could be linked to the reduced cellular energy levels. First, we showed that pollen grains from copper‐deficient plants accumulated a high level of ROS while supplementing pollen germination medium with an antioxidant, L‐ ascorbate, rescued pollen germination defect of copper‐deficient plants (Figure 4d and Figure S2). Second, COX activity was significantly reduced under copper deficiency in both rosette leaves and floral buds (Table 2). The reduction in COX activity results in over‐accumulation of O_2_ in the mitochondria matrix, which, in turn, leads to over‐production of ROS and consequent damage to cells. ROS accumulation under copper deficiency can also stem from the reduced Cu/Zn SOD activity (Ravet & Pilon, 2013). In addition, the reduction in COX activity leads to a reduction in cellular ATP production (Droppa et al., 1984). Consistently, *A. thaliana* COX11 homolog is involved in the insertion of copper into the COX complex during its assembly in mitochondria, is expressed in germinating pollen among other tissues, and its loss‐of‐function impairs pollen germination (Radin et al., 2015). Together, our results implicate an imbalance in the ROS level and the reduced energy level in copper‐deficiency mediated male infertility (Figure 8).

### 4.5 Copper deficiency leads to abnormal anther development

We also detected abnormality in anther morphology in copper‐deficient plants (Figure 5a). At the early stage of anther development (anther stage 5, floral stage 9), four cell layers are present in the anther wall including epidermis, endothecium, middle layer, and tapetum that is a nutritive tissue (Figure 5a‐I). At this stage, pollen mother cells (PMCs) are formed within anther locules. PMCs undergo meiosis to form microspores, which are then differentiated into the three‐cell pollen grains at stage 12 of anther and flower development (Sanders et al., 1999). Under copper deficiency, however, except for epidermis, the other three cell layers, including tapetum, were absent from the anther wall (Figure 5a‐V). These undefined structures remained throughout the middle and late stages of anther development in copper‐deficient anthers (Figure 5a‐VI and ‐VII).

We speculate that these defects in copper‐deficient flowers occur due to the significantly reduced expression of genes involved in anther cell determination (Figure 5b). Specifically, Arabidopsis, *BAM1* and *BAM2* (*Barely Any Meristem*) encode CLAVATA1‐related leu‐rich repeat receptor‐like kinases (LRR‐RLKs). The loss‐of‐function of both genes in the *bam1bam2* double mutant shows abnormal anther lacking the endothecium, middle, and tapetum layers (Hord et al., 2006). *RPK2* is also an LRR‐RLK which is required for early anther development (Mizuno et al., 2007). Anthers in the *rpk2* mutant lack the middle layer, have abnormal hypertrophic tapetal cells, as well as thickened and lignified endothecium cells, which together, lead to failure in pollen production and release. CIK1 to CIK4 (Clavata3 insensitive receptor kinase1 to 4) have shown to function as co‐receptors of BAM1 and BAM2 and RPK2 to control cell fate specification during early anther development in Arabidopsis (Cui et al., 2018). The loss‐of‐function of these four CIKs reduces fertility. Similarly, undefined structures were observed in gynoecium cross‐sections of the copper‐deficient flowers (Figure 5a‐VIII).

### 4.6 Copper deficiency‐based reduction in anther dehiscence is mediated, in part, *via* the reduced lignification, which in turn, may occur through conserved copper‐miRNAs

The expression of *copper‐microRNAs* including *miR397*, *miR398*, *miR408,* and *miR857*, is upregulated in roots and leaves of *A. thaliana* in response to copper deficiency to facilitate the turnover of non‐essential copper proteins and contribute to the copper economy (Pilon, 2017). Here, we show that copper microRNAs respond to copper deficiency differently depending on the stage of flower development. Specifically, *miR397a/b, miR398b/c*, and *miR857* were upregulated in floral buds but not in open flowers, while *miR398a* and *miR408* were upregulated in open flowers but not in floral buds. This observation suggests the unique roles of specific copper‐microRNAs in maintaining copper homeostasis during flower development.

Among well‐established copper microRNAs targets are copper proteins, *plantacyanin* and *laccases* (Pilon, 2017). Thus, it was not surprising that the expression of *plantacyanin* and all *LAC* genes tested was downregulated in flowers and primary inflorescence (Figure 6; Figure S3). Plantacyanins have a conserved copper‐binding site and are not essential for the growth and development of plants as evidenced by a lack of defects in *A. thaliana* and *O. sativa* plantacyanin mutants (Dong et al., 2005; Einsle et al., 2000; Ryden & Hunt, 1993; Zhang, Zhang, et al., 2018). Thus, the decrease in *plantacyanin* expression under copper deficiency is part of the copper economy mode aiming to channel copper from non‐essential to essential copper proteins.

Consistent with the role of *LAC4, LAC11,* and *LAC17* in lignin deposition in *A. thaliana* roots and stems (Zhao et al., 2013), the downregulation of their expression under copper deficiency (Figure 6b) was associated with the decreased lignin accumulation in anthers and primary inflorescence (Figure 6c; Figure S3). The decreased lignin accumulation was also observed in *A. thaliana* (*cv. Ler,* Figure S4f). Secondary wall thickening that includes lignification and cellulose deposition, occurs in the endothecium layer in anthers and is essential for anther dehiscence and pollen dispersal (Cecchetti et al., 2013; Hao et al., 2014a; Mitsuda et al., 2005; Yang et al., 2017). Thus, we propose that the entirely abolished anther dehiscence in copper‐deficient plants (Figure 6d) is associated with the loss of lignin in the endothecium and stomium region (Figure 6c) and is among contributing factors of the reduced fertility under copper deficiency. Lignin deposition was completely abolished from the interfascicular fibers and was only associated with the xylem (Figure S3). While interfascicular fibers are not essential for plant survival, their development and lignification are important for the stem strength and structural support of the plant body (Zhong et al., 1997). Failure to lignify this tissue results in the weakening of stems that ultimately can translate to crop lodging in the field (Broadley et al., 2012; Zhong et al., 1997).

### 4.7 The relationship between copper deficiency and leaf senescence

Natural leaf senescence is an important developmental process that ensures the remobilization of nutrients including minerals from the senescing tissues to developing reproductive organs and seeds (Leopold, 1961). Natural leaf senescence, typically triggered by the age of leaves, is associated with a transition to reproduction and can be initiated by hormones including jasmonic acid (JA) (Woo et al., 2019). Environmental stresses, as well as nutrient deficiency, are known to cause premature senescence (Leopold, 1961; Woo et al., 2019; Xie et al., 2014). While natural senescence increases reproductive success, premature senescence is often correlated with severely decreased yields (Leopold, 1961; Woo et al., 2019; Xie et al., 2014). We have shown recently that JA levels increase in leaves of copper‐deficient *A. thaliana* suggesting that deficiency for this mineral causes premature senescence, and could be among the reasons for dropped seed yield (Yan et al., 2017). On the other hand, we and others have shown that copper deficiency delays the transition to flowering (Figure 2) and thus, would be expected to delay senescence. Hill et al. (1978) have also shown that copper deficiency delays chlorophyll degradation of mature wheat leaves, further reinforcing the notion that unlike other mineral deficiencies that trigger senescence, copper deficiency might delay it.

To reconcile this discrepancy, we evaluated the effect of copper deficiency on the expression of senescence marker genes, *SAC12, SAG13,* and *WRKY53*. *SAG12* encodes a cysteine protease that is expressed only in senescing tissues. It is involved in nitrogen remobilization and its expression is upregulated by JA (He et al., 2002; James et al., 2018; Noh & Amasino, 1999). *SAG13* encodes a senescence‐associated protein that is also required for resistance against fungal pathogens. *SAG13* is induced during stresses such as oxidative stress and is required for the normal seed germination, seedling growth, and anthocyanin accumulation (Dhar et al., 2020). *WRKY53* is a master regulator of age‐induced leaf senescence that is associated with the onset of natural senescence and acts upstream in the senescence transcriptional cascade (Zentgraf et al., 2010). Both *SAG12* and *SAG13* are downstream WRKY53 targets with *SAG12* considered a marker for age‐dependent natural senescence while *SAG13* is commonly used to evaluate stress‐induced senescence (Zhao et al., 2018).

We found that copper deficiency mounted a distinct transcriptional response of senescence‐associated genes (Figure 7). Specifically, while the expression of *SAG12* was significantly upregulated by copper deficiency in both mature and young leaves, the expression of *SAG13* and *WRKY53* was significantly downregulated in leaves of copper‐deficient plants (Figure 7). Given that WKRY53 acts upstream in the transcriptional cascade leading to leaf senescence and its expression is downregulated under copper deficiency, we conclude that unlike other stress factors, copper deficiency delays the onset of senescence. This suggestion is also consistent with the delayed transition to flowering under copper deficiency (Figure 2). However, the question remains why *SAG12* that is also among WRKY53 targets is upregulated under copper deficiency and why its transcript level is nearly ninefold higher in young versus mature leaves in copper‐deficient versus copper‐sufficient plants. The increased expression of *SAG12* under copper deficiency may be triggered by JA, which, accumulates in leaves of copper‐deficient plants (He et al., 2002; Yan et al., 2017). It is also possible that a nutritionally‐derived, yet unidentified, signal triggers the upregulation of *SAG12* expression independently of WRKY53 to initiate nutrient remobilization from leaves to, eventually, ensure the transition to flowering. In this regard, copper deficiency is more pronounced in young leaves because of poor phloem‐based copper mobility (Broadley et al., 2012). Coincidentally, the *SAG12* expression is highly upregulated by the copper deficiency in young leaves, reinforcing the notion that nutritionally derived signal may exist to trigger the *SAG12* expression for stimulating protein degradation and amino acid utilization required for the growth of young leaves and subsequent transition to flowering.

To conclude, copper deficiency exerted pleiotropic effects on plant growth and development that include auxin‐related changes in shoot branching, increased flower bud growth, and aberrant stigma morphology with the latter contributing to the reduced gynoecium fertility (Figure 8). The reduced pollen fertility of copper‐deficient plants stemmed from multiple factors including the failure of anthers to dehisce and disperse pollen as well as the increased ROS level concomitant with the decreased cellular energy (Figure 8). The delayed transition to flowering observed in copper‐deficient *A. thaliana* possibly occurred through the decreased expression of *FT,* which, in turn, might have occurred *via* the reduced production of carbohydrates including the sucrose, the established *FT* activator (Figure 8). Copper deficiency also delayed senescence as evidenced by the significantly decreased expression of the master regulator of the onset of senescence, *WRKY53*. Copper deficiency triggered *SAG12* expression *via* the WRKY53‐independent pathway that, we hypothesize, involved the copper deficiency‐derived nutrient signaling pathway. Overall, this study opens several new areas for the in‐depth investigation into the relationship between copper homeostasis and hormone‐mediated shoot architecture, gynoecium fertility and copper deficiency‐derived signals leading to the delay in flowering time and senescence.

## CONFLICT OF INTEREST

The authors declare no conflict of interest.

## AUTHOR CONTRIBUTIONS

MRI received the concept and performed the experiments and analyzed the data; MRI and OKV wrote and edited the manuscript.

## Supporting information

Table S1‐Fig S1‐S4
