# Supplementary material for: Copper deficiency alters shoot architecture and reduces fertility of both gynoecium and androecium in Arabidopsis thaliana
Source: Plant Direct. 2020 Nov 29;4(11):e00288. doi: 10.1002/pld3.288 (PMC7700745; doi:10.1002/pld3.288)
Supplement: Supplementary file 1 — Table S1‐Fig S1‐S4 [file PLD3-4-e00288-s001.pdf]

Supplemental Tables and Figures

Supplemental Table S1. List of RT-qPCR primers and their sequences.

Gene ID	Primer name	Primer sequence (5'-3')
At3g18780	ACT2_F	GTCGTACAACCGGTATTGTGCTG
	ACT2_R	CCTCTCTCTGTAAGGATCTTCATGAG
At1g65480	FT_F	GTTGGTGACTGATATCCCTGCT
	FT_R	CGATGAATTCCTGCAGTGGGAC
At3g11435	miRNA172c_F	AGCTACTGTTCGCTGTTG
	miRNA172c_R	CAGCTGCAGCATCATCAAGA
At3g55512	miRNA172d_F	AGTCATTGTTTGCTATTGC
	miRNA172d_R	CCATTTAATTGCCGCTGCAGCATC
At5g65700	BAM1_F	CATAGTGAGGCTTCTCGGATTT
	BAM1_R	TGCAAGTGTCCTCCTTTCTTAC
At3g49670	BAM2_F	GCCAAGGGTCTTTGCTATCT
	BAM2_R	GTTGGAATCGAGGAGGATGTT
At3g02130	RPK2_F	TGAGACAAGGAAGGGCAAAG
	RPK2_R	CCGTACAAACAACCGCTAAATG
At1g60800	CIK1_F	GTTGCGCTACTCTGCACTCA
	CIK1_R	TCACCAGTACCGTTCTGCGTC
At2g23950	CIK2_F	GGACTGGAACACAAGGAAGAA
	CIK2_R	GCCTTGACATCTCGGTGAATA
At4g30520	CIK3_F	GGATCGAGAACTCGGAACTAAC
	CIK3_R	GACGATGAGCTGGCAGATATT
At5g45780	CIK4_F	CACAGCCACATCCGAATCTA
	CIK4_R	CTAGCTTCATACCCTCCTTCAC
At4g13555	miRNA397b_F	TGAATGAACATCATTGAGTGC
	miRNA397b_R	ACATAATTGAATGCAACGCTG
At2g03445	miRNA398a_F	GAAATTTCAAAGGAGTGGCA
	miRNA398a_R	ATTCAAAGGGGTGACCTGA
At5g14545	miRNA398b/c_F	GGATCTCGACAGGGTTGATATG
	miRNA398b/c_R	AAGAGCTCAGCAGGGGTG
At2g47015	miRNA408_F	AGACAAAGCGGTAATGAGAGAG
	miRNA408_R	TCCATTGAAAGCTGTGAAATGAAAGG
At4g13554	miRNA857a_F	ACCTAAAGGTAGCGTGACTATTG
	miRNA857a_R	TCCGATTCCTACAATACACCTTC
At2g02850	Plantacyanin_F	ACAGGGGCAAAACCTTACACTTCAG
	Plantacyanin_R	CATATCGCTTTCGCAATGGTTTGGA
At1g18140	LAC1_F	AGTCGTAGCTCTCCCGAATA
	LAC1_R	GTCTATCTTCGTCGGGACTTTAC
At2g38080	LAC4_F	CTTCAAGATACCGGCGTCATAG
	LAC4_R	TTGGGTCTTTCGTGGAGTTG
At3g09220	LAC7_F	CTATCGATCTTGGAAGCAGTGT
	LAC7_R	TACGTTCGGGTTCGTGTAATC

Supplemental Table S1 continues

At5g01040	LAC8_F	CATGCACCTCCACGGTTTCAACTT
	LAC8_R	GGTGGTACACCAACGGTATTAT
At5g60020	LAC17_F	CATCGCAAATCACACGGTTAC
	LAC17_R	GTCTGACCAGGAGCAATTAAGA
At5g45890	SAG12_F	GGCGAATCTACTAACGGATCAA
	SAG12_R	TTCATGGCAAGACCACATAGT
At2g29350	SAG13_F	GCTTTCCATCTCTCACAGCTTGCC
	SAG13_R	GCCAGCTGATTCATGGCTCCTTTG
At4g23810	WRKY53_F	TTTGCCGATGGAGGAGGTTCTAGC
	WRKY53_R	GCCTCTCTCTGGGCTTATTCTCAC

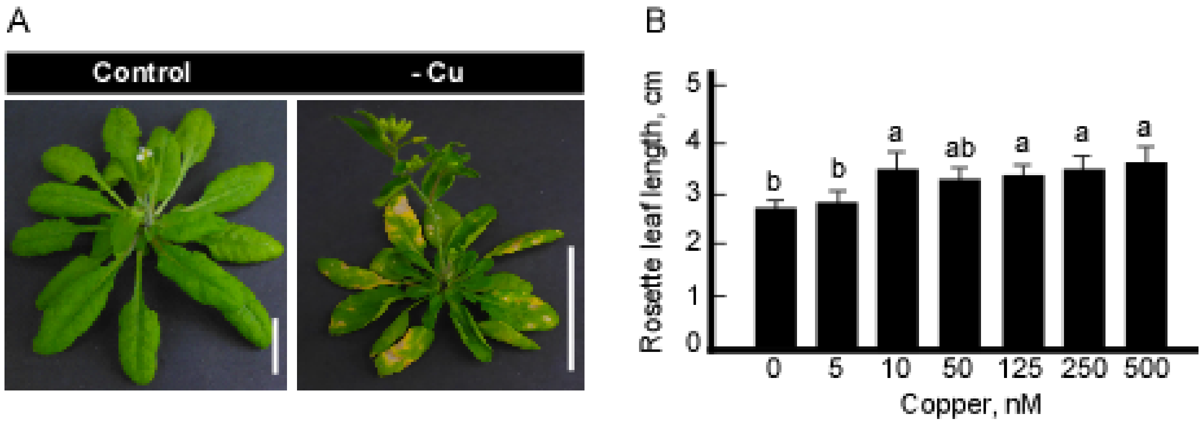

Supplemental Fig. S1. Copper deficiency leads to a small stature in *Arabidopsis*.

(A) Shows representative images of plants grown hydroponically with or without 250 nM $CuSO_4$ at flowering. Scale bar = 1 cm. (B) Rosette leaves length is shown upon transition to flowering for plants grown hydroponically under indicated copper concentrations. Rosette leaves were measured upon transition to flowering for each plant. Values are means ± SE (n = 3 independent experiments with 5-10 plants analyzed in each experiment). Levels not connected by same the letter are significantly different ($P < 0.05$) determined by the Tukey-Kramer HSD test using JMPPro 14.

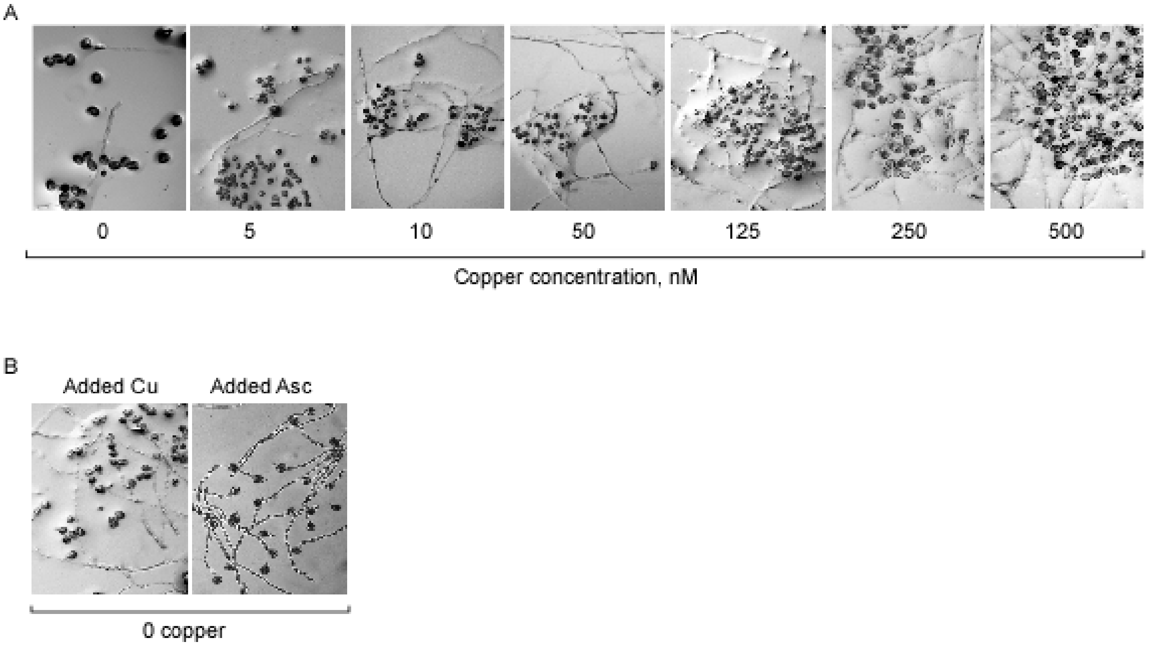

Supplemental Fig. S2. Representative images of *in vitro* pollen germination.

(A) Shows representative images of pollen germination for plants grown hydroponically in different copper concentrations as indicated. (B) Shows representative images of pollen germination for plants grown hydroponically without copper until flowering. Copper (20 nM) or L-ascorbate (5 μM) were then added directly to the pollen germination media. Note that the application of copper or L-ascorbate rescued the pollen germination defect of copper-deficient plants.

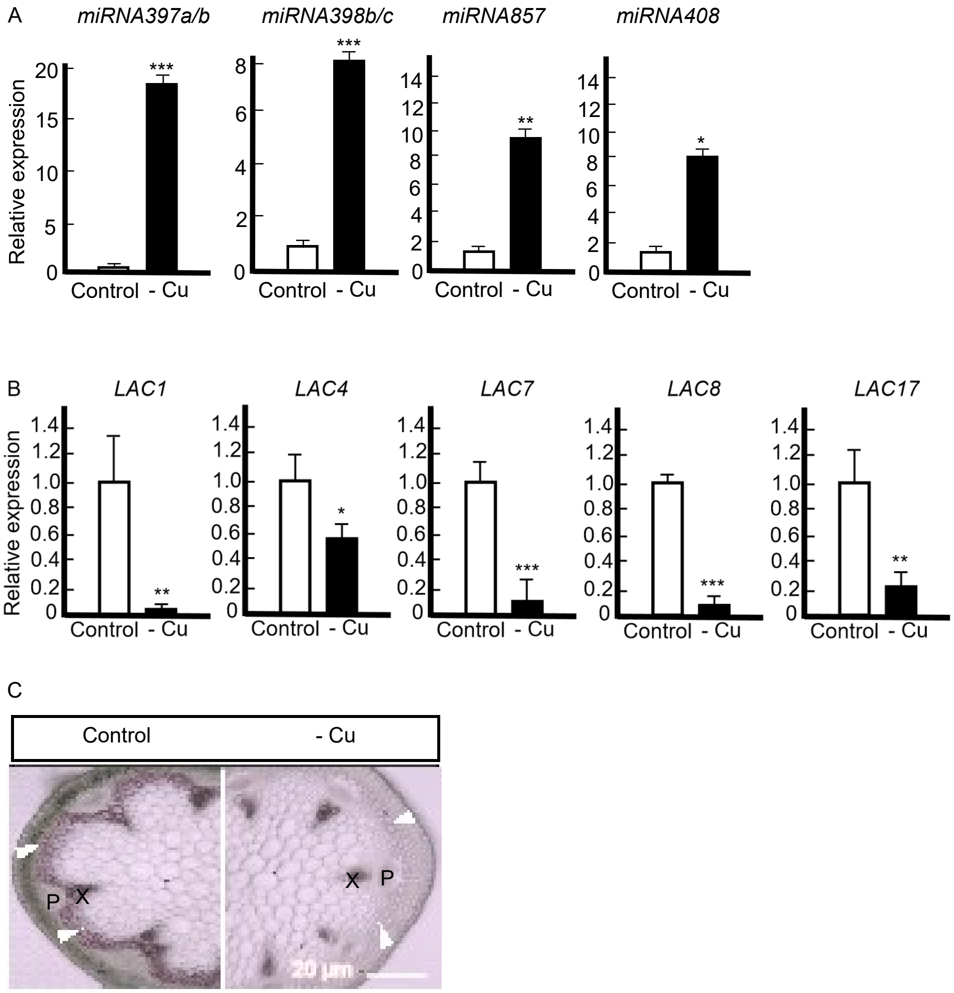

Supplemental Fig. S3. Copper deficiency decreases lignin biosynthesis in primary inflorescence through accumulation of *copper-miRNAs*.

(A) Shows the increase in the transcript abundances of *copper-miRNAs* in inflorescence stem under copper deficiency. (B) Shows the decrease in the transcript abundances of lignin biosynthesis genes, *laccases*, under copper deficiency. In (A) and (B), plants were grown hydroponically with or without 250 nM $CuSO_4$ until flowering. Values are means ± SE (n = 3 independent experiments with at least three to five plants analyzed in each experiment). Asterisks (*, $P < 0.05$); **, $P < 0.001$ and ***, $P < 0.001$ defined by Student's *t*-test) indicate statistically significant differences compared to control condition. (C) Cross section of primary inflorescence showing lignin deposition pattern. Phloroglucinol-HCl stains lignin in red. Representative images are presented here from three independent experiments, each with at least 10 plants per condition. P and X refer to phloem and xylem, respectively. White arrowheads show sites of lignin deposition in the interfascicular xylem fibers which is abolished under copper deficiency condition.

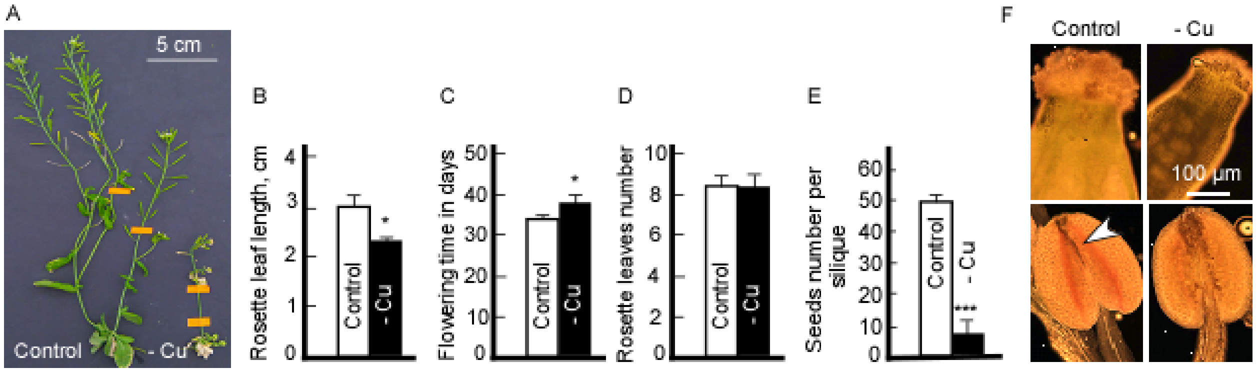

Supplemental Fig. S4. *Landsberg erecta* (*Ler*) response to copper deficiency.

(A) Shows representative images of plants grown hydroponically with or without 250 nM $CuSO_4$ for eight weeks. Scale bar = 5 cm. Rosette leaf length (B), flowering time (C), and number of rosette leaves (D) are shown upon transition to flowering for plants grown hydroponically with or without 250 nM $CuSO_4$. Rosette leaves were measured upon transition to flowering for each plant in (B). (E) Seeds number per silique are shown for plants grown hydroponically with or without 250 nM $CuSO_4$. (F) Shows representative images of the stigma and anther from plants grown hydroponically with or without 250 nM $CuSO_4$. Note that stigma from a copper-deficient plant is almost papilla-less. Phloroglucinol-HCl was used to stain lignin in anther. Lignin stains red. Note that a copper-deficient anther lacks lignin in the stomium region (white arrowhead). Scale bar = 100 µm. In (B) to (E), values are means ± SE (n = 3 independent experiments with 5-10 plants analyzed in each experiment). Asterisks (*, $P < 0.05$; ***, $P < 0.001$ as determined using Student's *t*-test) indicate statistical significance difference compared to control condition.
